# Supplementary material for: Sorting at embryonic boundaries requires high heterotypic interfacial tension
Source: Nat Commun. 2017 Jul 31;8:157. doi: 10.1038/s41467-017-00146-x (PMC5537356; doi:10.1038/s41467-017-00146-x)
Supplement: Supplementary file 2 — Supplementary Software 1 [file 41467_2017_146_MOESM2_ESM.zip › PottsModel/SrcPottsModel/doc/index-files/index-21.html]

V-Index


JavaScript is disabled on your browser.


Skip navigation links


- Overview
- Package
- Class
- Use
- Tree
- Deprecated
- Index
- Help

- Prev Letter
- Next Letter

- Frames
- No Frames

- All Classes

A B C D E F G H I J K L M N O P R S T U V W 


## V

valueOf(String) - Static method in enum engine.CellShapeCSVLabel
:   Returns the enum constant of this type with the specified name.

valueOf(String) - Static method in enum engine.PottsEngine.State
:   Returns the enum constant of this type with the specified name.

valueOf(String) - Static method in enum gui.PixelShape.Edge
:   Returns the enum constant of this type with the specified name.

valueOf(String) - Static method in enum gui.PixelShape.Type
:   Returns the enum constant of this type with the specified name.

valueOf(String) - Static method in enum gui.PottsFrame.Action
:   Returns the enum constant of this type with the specified name.

valueOf(String) - Static method in enum model.Cell.CellType
:   Returns the enum constant of this type with the specified name.

valueOf(String) - Static method in enum model.Cell.Energy
:   Returns the enum constant of this type with the specified name.

valueOf(String) - Static method in enum model.Cell.State
:   Returns the enum constant of this type with the specified name.

valueOf(String) - Static method in enum model.Constants.Model
:   Returns the enum constant of this type with the specified name.

valueOf(String) - Static method in enum model.EnergyFunction.Energy
:   Returns the enum constant of this type with the specified name.

valueOf(String) - Static method in enum model.IPositionManager.Position
:   Returns the enum constant of this type with the specified name.

valueOf(String) - Static method in enum model.Lattice.State
:   Returns the enum constant of this type with the specified name.

valueOf(String) - Static method in enum model.Lattice.Type
:   Returns the enum constant of this type with the specified name.

valueOf(String) - Static method in enum model.Pixel.PixelType
:   Returns the enum constant of this type with the specified name.

values() - Static method in enum engine.CellShapeCSVLabel
:   Returns an array containing the constants of this enum type, in
    the order they are declared.

values() - Static method in enum engine.PottsEngine.State
:   Returns an array containing the constants of this enum type, in
    the order they are declared.

values() - Static method in enum gui.PixelShape.Edge
:   Returns an array containing the constants of this enum type, in
    the order they are declared.

values() - Static method in enum gui.PixelShape.Type
:   Returns an array containing the constants of this enum type, in
    the order they are declared.

values() - Static method in enum gui.PottsFrame.Action
:   Returns an array containing the constants of this enum type, in
    the order they are declared.

values() - Static method in enum model.Cell.CellType
:   Returns an array containing the constants of this enum type, in
    the order they are declared.

values() - Static method in enum model.Cell.Energy
:   Returns an array containing the constants of this enum type, in
    the order they are declared.

values() - Static method in enum model.Cell.State
:   Returns an array containing the constants of this enum type, in
    the order they are declared.

values() - Static method in enum model.Constants.Model
:   Returns an array containing the constants of this enum type, in
    the order they are declared.

values() - Static method in enum model.EnergyFunction.Energy
:   Returns an array containing the constants of this enum type, in
    the order they are declared.

values() - Static method in enum model.IPositionManager.Position
:   Returns an array containing the constants of this enum type, in
    the order they are declared.

values() - Static method in enum model.Lattice.State
:   Returns an array containing the constants of this enum type, in
    the order they are declared.

values() - Static method in enum model.Lattice.Type
:   Returns an array containing the constants of this enum type, in
    the order they are declared.

values() - Static method in enum model.Pixel.PixelType
:   Returns an array containing the constants of this enum type, in
    the order they are declared.

Vector - Interface in model


VectorTests - Class in model


VectorTests() - Constructor for class model.VectorTests


VectorUtils - Class in model


VectorUtils() - Constructor for class model.VectorUtils

A B C D E F G H I J K L M N O P R S T U V W

Skip navigation links


- Overview
- Package
- Class
- Use
- Tree
- Deprecated
- Index
- Help

- Prev Letter
- Next Letter

- Frames
- No Frames

- All Classes
